# Mechanically Robust Flexible Multilayer Aramid Nanofibers and MXene Film for High-Performance Electromagnetic Interference Shielding and Thermal Insulation

**DOI:** 10.3390/nano11113041

**Published:** 2021-11-12

**Authors:** Jun Zhou, Junsheng Yu, Dongyu Bai, Huili Liu, Lu Li

**Affiliations:** 1 State Key Laboratory of Electronic Thin Films and Integrated Devices, School of Optoelectronic Science and Engineering, University of Electronic Science and Technology of China (UESTC), Chengdu 610054, China; along_zj@163.com (J.Z.); jsyu@uestc.edu.cn (J.Y.); 2 Chongqing Key Laboratory of Materials Surface & Interface Science, School of Materials Science and Engineering, Chongqing University of Arts and Sciences, Chongqing 402160, China; 3 School of Chemistry & Chemical Engineering, Chongqing University, Chongqing 400044, China; liuhl0827@163.com; 4 Chongqing Key Laboratory of Environmental Materials and Remediation Technologies, College of Chemistry and Environmental Engineering, Chongqing University of Arts and Sciences, Chongqing 402160, China

**Keywords:** multilayer structure, MXene, ANF, electromagnetic interference shielding, thermal insulation

## Abstract

In order to overcome the various defects caused by the limitations of solid metal as a shielding material, the development of electromagnetic shielding materials with flexibility and excellent mechanical properties is of great significance for the next generation of intelligent electronic devices. Here, the aramid nanofiber/Ti_3_C_2_T_x_ MXene (ANF/MXene) composite films with multilayer structure were successfully prepared through a simple alternate vacuum-assisted filtration (AVAF) process. With the intervention of the ANF layer, the multilayer-structure film exhibits excellent mechanical properties. The ANF2/MXene1 composite film exhibits a tensile strength of 177.7 MPa and a breaking strain of 12.6%. In addition, the ANF5/MXene4 composite film with a thickness of only 30 μm exhibits an electromagnetic interference (EMI) shielding efficiency of 37.5 dB and a high EMI-specific shielding effectiveness value accounting for thickness (SSE/t) of 4718 dB·cm^2^ g^−1^. Moreover, the composite film was excellent in heat-insulation performance and in avoiding light-to-heat conversion. No burning sensation was produced on the surface of the film with a thickness of only 100 μm at a high temperature of 130 °C. Furthermore, the surface of the film was only mild when touched under simulated sunlight. Therefore, our multilayer-structure film has potential significance in practical applications such as next-generation smart electronic equipment, communications, and military applications.

## 1. Introduction

With the rapid development of electronic technology, people’s lives have become more prosperous, and the accompanying electromagnetic interference (EMI) and radiation will not only affect normal working electronic equipment but also pose a potential threat to human health [1,2,3]. Traditional EMI shielding materials are usually made of metals with the high electrical conductivity, but the widespread application of traditional metal EMI shielding materials is hindered by their stiffness, high density and poor anticorrosive performance. In recent years, the vigorous development of flexible electronics has promoted the emergence of various conductive polymer composite EMI shielding materials due to their easy processability and corrosion resistance. Such composite material are composed of polymer matrix and conductive fillers such as carbon nanotubes [4,5,6], graphene [7,8,9] and silver nanowires [10,11,12]. However, large amounts of fillers were added into polymer matrix in order to obtain the ideal EMI shielding performance, which depressed the flexibility and mechanical performance [13,14]. Therefore, it is still a huge challenge to develop a high-efficiency EMI shielding flexible material with low thickness and excellent mechanical properties.

As a new class of two-dimensional inorganic compounds, MXenes have attracted extensive attention in recent years [4,15]. MXenes are composed of transition metal carbides, nitrides or carbonitrides with a thickness of several atomic layers. They exhibit the metal conductivity and hydrophilicity of transition metal carbides, owing to the hydroxyl or terminal oxygen on the surface [16,17]. Therefore, MXenes have been reported for application in various fields, such as supercapacitors [18,19], energy storage [20,21], smart wear [22,23], electromagnetic shielding [24,25,26,27], etc. Gogotsi et al. first reported that MXene with sodium alginate (SA) was used to prepare a high-efficiency EMI shielding film with remarkable conductivity and multiple internal reflections between MXene layers [4]. An absorption-based porous MXene/polyvinyl alcohol (PVA) composite foam was prepared through a freeze-drying method with a shielding effectiveness (SE) of 5136 dB·cm^2^ g^−1^ [28]. Similarly, owing to the porous structure for highly efficient wave attenuation, Zhao et al. assembled a three-dimensional (3D) aerogel using MXene and graphene oxide presenting an electromagnetic shielding effectiveness of 50 dB under X band [29]. However, the poor mechanical properties of MXene impede its extensive application in fields such as artificial intelligence, military aerospace, smart wear, etc.

Recently, different polymers or nanofibers have been introduced to MXene through rich groups on the surface of MXene and polymers or nanofibers to improve its overall mechanical properties [10,30,31,32,33,34]. Sun et al. constructed a nacre-like brick-and-mortar microstructure film composed of MXene and xanthan gum, showing a strong tensile strength of about 116.48 MPa while maintaining high conductivity, which was much higher than that of pure MXene film [35]. A composite film composed of MXene and cellulose nanofibers (CNF) with excellent mechanical properties and great EMI shielding performance (~40 dB) was fabricated by the vacuum-assisted filtration method [36]. Nanofiber could significantly reinforce the properties of the composite. [37,38] As a one-dimensional nanofiber, aramid nanofiber (ANF) presents ultra-high strength, high modulus, large specific surface area and excellent stability due to the strong interactions between molecular chains such as hydrogen bonds, van der Waals forces and high crystallinity [39,40,41,42,43,44].

In this work, the ultra-thin flexible ANF/MXene multilayer-structure nanocomposite film with strong mechanical properties was prepared via a simple alternate vacuum-assisted filtration (AVAF) method. The film with a hierarchical nanostructure was tightly bonded via the wide-bonding groups between the different layers, effectively avoiding contact of MXene with air. The ANF/MXene series film shows similar electromagnetic shielding performance, as well as better mechanical tensile properties, to the commonly used laminated film composed of CNF and MXene. As a result, the ANF5/MXene4 film exhibited EMI SE of 37.5 dB and specific shielding effectiveness values accounting for thickness (SSE/t) of 4718 dB·cm^2^ g^−1^ at a thickness of 30 μm. Additionally, the tensile strength of the multilayer ANF2/MXene1 film exhibited tensile strength of 177.7 MPa. In addition, multilayer films of ANF2/MXene1 with a thickness of 100 μm exhibit great thermal insulation properties under an electric field. The EMI shielding multifunctional composite film with multilayer alternating structure could be applied to many scenarios, such as aerospace, flexible electronics, and communications.

## 2. Experimental Section

### 2.1. Materials

Ti_3_AlC_2_ (MAX) powder (400 mesh) was purchased from Jinlin 11 Technology Co., Ltd., Jinlin, China. Kevlar 49 fiber was purchased from DuPont, Willmington, DE, USA. Potassium hydroxide (KOH) and hydrochloric acid (HCl, 36–38%) were purchased from Sinopharm Chemical Reagent Co., Ltd., Shanghai, China. Dimethyl sulfoxide (DMSO) and ethanol were obtained from Aladdin Reagent, Shanghai, China. Lithium fluoride (LiF) was supplied by Sigma Aldrich, St. Louis, MO, USA.

### 2.2. Preparation of ANF Dispersion

Kevlar 49 fiber (1.0 g) and KOH (1.5 g) were dissolved in DMSO (500 mL) at room temperature. The suspension was stirred magnetically for 7 days to obtain dark red ANF solution. Then, 50 mL of the deep red solution was added to 200 mL of deionized water with continuous stirring until the color of the solution became pale yellow. Afterwards, the colloidal ANF obtained by filtration was added to 100 mL deionized water, and the ANF solution was obtained after ultrasonication for 10 min and continuous stirring for 1 h.

### 2.3. Preparation of MXene Nanosheet Suspension

The MXene nanosheet suspension was prepared by a typical etching method, as reported [45]. Specifically, LiF (1 g) was dissolved in 9 mol/L HCl (20 mL) in a polytetrafluoroethylene (PTFE) beaker. Then, Ti_3_AlC_2_ (1.0 g) powder was slowly added into the PTFE beaker with a reaction temperature of 35 °C for 24 h under continuous stirring. The obtained mixture, Ti_3_C_2_T_x_, was washed with deionized water via centrifugation at 4500 rpm until the pH of the solution was about 6.0. After sonication at 160 W for 30 min and centrifugation at 4500 rpm for one hour, an upper black-green water solution with MXene was obtained.

### 2.4. Preparation of Multilayer-Structure ANF/MXene Films

The multilayer-structure ANF/MXene films were prepared using an unsophisticated vacuum-assisted filtration method [46]. For brevity, multilayer-structure films were marked as ANF (*n* + 1)/MXene (*n*), *n* + 1 and *n*, respectively, representing the number of layers of ANF and MXene in the film. The bottoms and tops of the films were composed of ANF layers to protect MXene from oxidation. Firstly, 10 mL of MXene aqueous suspension (5 mg/mL) and 50 mL of ANF dispersion (1 mg/mL) were divided equally into *n* parts and *n* + 1 parts. After that, ANF dispersion and MXene aqueous suspension were vacuum filtrated alternately onto a polyethersulfone membrane (pore diameter, 220 nm). Next, the remaining parts of MXene and ANF suspension were filtrated in turn until all were drawn. Finally, the resultant multilayer-structure films were completely dried under hot pressing at 70 °C with 1.2 MPa and peeled off from the membrane carefully. A series of multilayered ANF/MXene nanocomposite films were fabricated. For the convenience of comparison, the mixed MXene/ANF aqueous solution containing 10 mL of MXene aqueous suspension (5 mg/mL) and 50 mL of ANF dispersion (1 mg/mL) was prepared, and the homogeneous MXene/ANF film was formed by filtration and hot pressing in the same way. The pure MXene and ANF films with the same MXene (10 mL) ANF (50 mL) contents were fabricated in the same way.

### 2.5. Characterizations

All the morphologies and microstructures were observed on a field emission SEM (GeminiSEM 300, ZEISS, Jena, Germany) and transmission electron microscope (TEM, JEOL JEM-2100 F, Akishima, Japan). The specimens were quickly fractured after being immersed in liquid nitrogen for 1 h. X-ray diffraction (XRD, TD-3500, Dandong, China) patterns were used to characterize the partial structure and morphology of the films. X-ray photoelectron spectroscopy patterns (XPS Thermo Fisher K-Alpha, Parma, OH, USA) were used to analyze elemental composition. The resistance of the composite film was measured with a digital multimeter (DT9919, Chengdu, China). Electrical conductivities were calculated by the formula:

$$\sigma =\frac{L}{SR}$$

where *L*, *S* and *R* represent the length, cross-sectional area and resistance of the test sample, respectively. The mechanical test of the composite films was performed by a dynamic mechanical testing machine (Test Star, WANCE, Shenzhen, China) with a rate of 5 mm/min. A vector network analyzer (Agilent PNA-N5244A, Santa Clara, CA, USA) was used to analyze the EMI SE of composite films in the frequency ranges of 8.2–12.4 GHz (X-band). By recording the scattering parameters, *S*_11_ and *S*_21_, to calculate the power coefficients of reflection (*R*), transmission (*T*) and absorption (*A*), the specific calculation method of $SE_{T}$, $SE_{R}$, $SE_{A}$ was summarized by the following Equations:

(1) $$R=|S_{11}{|}^{2}=|S_{22}{|}^{2}\;T=|S_{21}{|}^{2}=|S_{21}{|}^{2}$$

(2) A+R+T=1

(3) $$SE_{R}=-10\log\left(1-R\right)\;\;SE_{A}=-10\log\left(\frac{T}{1-R}\right)$$

(4) $$SE_{T}=SE_{R}+SE_{A}+SE_{M}$$

(In particular, $SE_{M}$ could be ignored when the $SE_{T}$ exceeds 15 dB) [47].

## 3. Results and Discussion

**Figure 1:**
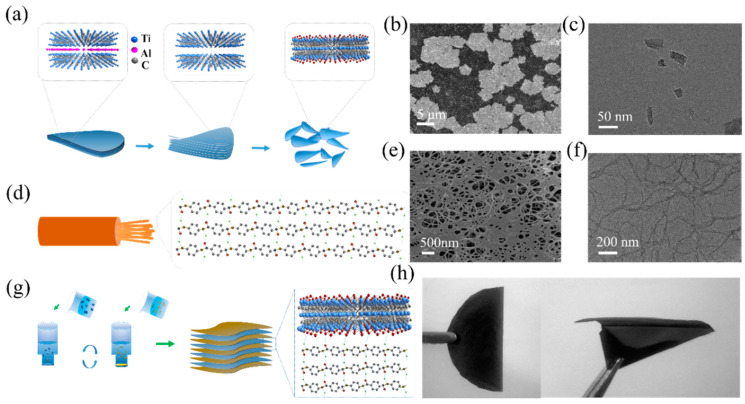
(**a**) Schematic illustration of the synthesis of Ti_3_C_2_T_x_ by chemical etching; (**b**,**c**) SEM and TEM images of d-Ti_3_C_2_T_x_; (**d**) preparation of ANF dispersion by deprotonation; (**e**,**f**) SEM and TEM images of ANFs; (**g**) preparation of ANF/MXene multilayer-structure films by vacuum-assisted filtration; (**h**) digital image of multilayer-structure film under folding.

Figure 1a specifically shows the preparation of Ti_3_C_2_T_x_ MXene nanosheets from the MAX phase by selectively etching with HCl/LiF system. The multilayer Ti_3_C_2_T_x_ (m-Ti_3_C_2_T_x_) with “accordion” structure was obtained after selective etching of Ti_3_AlC_2_ (MAX) (Figure S1) and subsequently transformed into few-layer and single-layer Ti_3_C_2_T_x_ (d-Ti_3_C_2_T_x_) nanosheets after further ultrasonic delamination (Figure 1b,c). The direct change of the crystal structure of Ti_3_C_2_T_x_ could be observed from the XRD pattern (Figure S2). The diffraction peaks, (101), (103), (104) and (105), were greatly reduced or disappeared, and the (002) peak shifted to the left from 9.7° to 6.3°, which proved the elimination of aluminum, indicating the successful preparation of Ti_3_C_2_T_x_ nanosheets. X-ray photoelectron spectroscopy (XPS) was used to analyze the chemical characteristics of the d-Ti_3_C_2_T_x_ surface, showing that many functional groups (–O, –OH,) were introduced on the surface of d-Ti_3_C_2_T_x_ under etching (Figure S3). ANF dispersion was prepared by a deprotonation method (Figure 1d). The addition of Kevlar fiber to the KOH/DMSO system reduced the interchain interaction between molecular chains by protonation under the action of KOH, which facilitated the transition from large-scale Kevlar fibers to many micro-ANFs to obtain individual polymer chains. Figure S4 illustrates the transformation process of ANF nanofibers in 7 days. From the SEM and TEM images of ANF (Figure 1e,f), the nanofibers were about 10 nm in diameter and a few microns in length after deprotonation. The Tyndall effect diagrams of Ti_3_C_2_T_x_ and ANF solutions are shown in Figure S5, revealing their good dispersibility in water.

Figure 1g presents a brief overview of the preparation process of the multilayer-structure ANF/MXene films. All MXene layers were protected by the ANF layer so that they would not be oxidized due to exposure to outside air, while the ANF layer in the multilayer-structure film could be used as a support layer, increasing the mechanical properties of the composite film. In Figure 1h, the multilayer-structure film is shown to be easily folded in half or even folded into the desired complex shape (aircraft) without damage, indicating the excellent flexibility.

### 3.1. Microstructures of Alternating Laminated ANF/MXene Films

**Figure 2:**
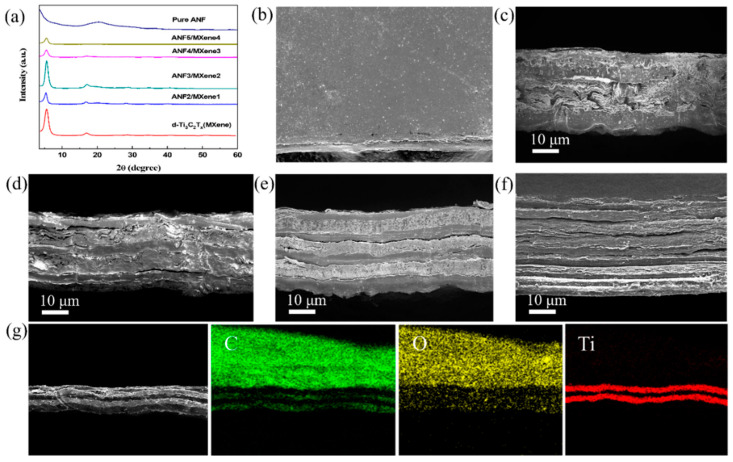
(**a**) XRD patterns of pure ANF, MXene and ANF/MXene multilayer-structure films; (**b**) SEM image of MXene1 film surface; (**c**–**f**) SEM images of cross-sections of films with different layers; (**g**) Elemental analysis of the cross-section of ANF3/MXene2 film.

Figure 2a shows the XRD patterns of pure ANF film, Ti_3_C_2_T_x_ MXene film and multilayer-structure ANF/MXene films. The typical, characteristic diffraction peak of ANF at around 20.5° was clearly observed, corresponding to (110). The surface of the multilayer film ANF2/MXene1 was uniform and flat (Figure 2b), indicating that the MXene layer was well protected in the inner layer. Figure 2c–f illustrates the SEM cross-sections of multilayer-structure films with different layers. The thickness of the MXene layer and the ANF layer of the composite film varied from 3 to 9 μm under different numbers of alternating layers. The internal composition of the conductive MXene layer and the mechanically strong ANF substrate layer led to high conductivity inside MXene and high insulation on the ANF substrate side of the film, facilitating excellent EMI performance and mechanical properties [48,49]. In contrast, when MXene and ANF randomly dispersed in the homogeneous MXene/ANF film (Figure S6), the electrical and mechanical properties of the film were affected by their distribution. An elemental energy spectrum analysis of ANF3/MXene2 film was performed in order to further demonstrate the layered structure of the multilayer-structure film. As seen in Figure 2g, the Ti, C and O elements were selectively distributed on the multilayer-structure film, where the distribution of Ti elements almost corresponded to the MXene layer in the SEM part. Moreover, basically no Ti element was observed between the MXene layer, indicating the integrity of the multilayer structure.

### 3.2. Mechanical Properties of Multilayer-Structure ANF/MXene Films

**Figure 3:**
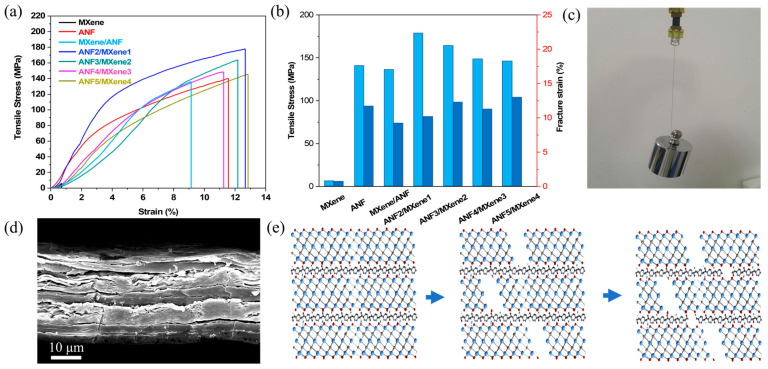
(**a**,**b**) Tensile-stress curves and statistics of pure ANF, MXene, homogeneous MXene/ANF film and multilayer-structure films; (**c**) digital image of ANF3/MXene2 film stretched by 500 g weight; (**d**) SEM images of cross-sections of ANF3/MXene2 film after tensile fracture; (**e**) Schematic illustrations of the cross-section of the multilayer-structure film after being subjected to mechanical tensile fracture.

Figure 3a, b exhibits the tensile stress-strain curve and mechanical properties of the multilayer films. As expected, the pure MXene film presented extremely low tensile strength (6.0 MPa) and fracture strain (0.69%), consistent with the previous reports (about 5.0 MPa and 1%, respectively) [34,50]. Meanwhile, the pure ANF film showed a very high tensile strength of 140.1 MPa and a breaking strain of 11.5%. The multilayer-structure ANF/MXene films exhibited better mechanical properties with the addition of ANF. As for ANF2/MXene1, the tensile strength and the breaking strain increased to 177.7 MPa and 12.6%, respectively. However, with the further increase in the number of MXene layers, the tensile strength and breaking strain of the multilayer-structure films gradually decreased. As a comparison, the mechanical properties of the homogeneous mixed MXene/ANF film were tested, showing a tensile strength of 135.85 MPa and a breaking strain of 9.14%. The comparison with other electromagnetic shielding materials is shown in Table S1. Figure 3c illustrates a digital image of ANF3/MXene2 film under the tensile force of 500 g of weight. Even under continuous shaking, the film also withstood the weight well, without breaking. The cross-sectional SEM image of ANF3/MXene2 film after being stretched is shown in Figure 3d. The hierarchical crack morphology could be clearly observed. Specifically, there were small debris and cracks between the MXene layers. Herein, a crack propagation model of multilayer-structure ANF/MXene films was proposed, as illustrated in Figure 3e. The MXene nanosheets would first begin to break with the tensile force, owing to the poor interlamination interaction, and the bonding group (hydrogen bond) between the MXene layer and the ANF layer was gradually destroyed [51], which resulted in cracks and fractures in the MXene layer. However, due to the existence of an ANF layer with strong mechanical properties, the entire structure of the alternating film was able to maintain its integrity. As the stretching continued, the ANF layer, as the load-bearing frame, slowly reached its limit, and the crack continued to expand until the alternating film was completely fractured. What’s more, due to the multilayer laminated structure, the film appeared to undergo obvious delamination during the fracture process.

### 3.3. Electromagnetic Shielding Performances of Multilayer-Structure ANF/MXene Films

**Figure 4:**
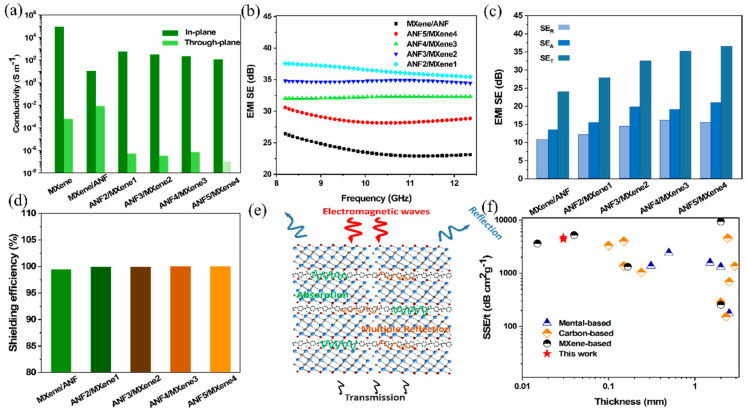
(**a**) Electrical conductivities of MXene film, homogeneous MXene/ANF film and multilayer-structure films; (**b**) EMI shielding performances of homogeneous MXene/ANF film and multilayer-structure films; (**c**) average EMI microwave reflection (*SE_R_*), microwave absorption (*SE_A_*) and total shielding effectiveness (*SE_T_*) of multilayer-structure films; (**d**) overall shielding efficiency of homogeneous MXene/ANF film and multilayer-structure films of ANF2/MXene1, ANF3/MXene2, ANF4/MXene3 and ANF5/MXene4; (**e**) schematic illustration of the electromagnetic shielding mechanism of multilayer-structure films; (**f**) comparison of SSE/t as a function of thickness with previous reports. The detailed information is listed in Table S1.

The electrical conductivity and thickness of the material are the key to determining the performance of the electromagnetic shielding material [52,53,54]. As shown in Figure 4a, the emerging 2D material MXene film exhibited a high electrical conductivity of 83,201 S m^−1^ due to its excellent electron-transmission ability [4,55]. With the increasing of the film layers, the conductivity of the films decreased slightly from 546.4 S m^−1^ to 116.9 S m^−1^. Due to the special multilayer structure of the alternating film designed in this work, the anisotropy in the vertical and horizontal directions led to horizontal conductivity 8–9 orders of magnitude higher than that in the vertical direction, which provides a new idea for the design of insulating electromagnetic shielding materials.

As expected, the existence of MXene nanosheets endows composite film with excellent EMI shielding performance. The EMI shielding performance of the multilayer-structure films was tested in the X band (8.2–12.4 GHz) according to the S-parameter calculation method [47]. In order to clarify the EMI shielding mechanism, Figure 4b,c shows the total EMI shielding efficiency (*SE_T_*), microwave reflection (*SE_R_*) and microwave absorption (*SE_A_*) calculated by Equations (3) and (4). The average EMI *SE_T_* of the homogeneous mixed MXene/ANF film was 23.8 dB, where *SE_A_* and *SE_T_* were 10.7 dB and 13.1 dB, respectively. Only a small part of the electromagnetic waves was able to penetrate, basically meeting the requirements of shielding materials. In contrast, the multilayer-structure films presented a substantially improved EMI shielding performance (27.7–37.5 dB), attributed to the continuous conductive network formed by the MXene nanosheets in each layer of the internal laminated structure. It was found that as the number of ANF and MXene layers increased, *SE_A_* and *SE_R_* presented a similar trend to *SE_T_*, while *SE_A_* affected EMI shielding performance more than *SE_R_*, which can be ascribed to the laminated structure and efficient conductive network of MXene nanosheets. Moreover, the increase in the layers also caused multiple reflections inside the material, improving the absorption of electromagnetic waves. Thus, the low microwave reflection and strong microwave absorption in the multilayer-structure composite film indicates an absorption-based shielding mechanism. Figure 4d reveals the shielding efficiency of homogeneous MXene/ANF film and multilayer-structure films. The multilayer-structure films showed an excellent shielding efficiency (above 99%). In particular, the ANF5/MXene4 film could block 99.99% of incident radiation, and only 0.01% of electromagnetic waves were able to pass through. The underlying mechanism for the multilayer-structure composite film is illustrated in Figure 4e. When the electromagnetic wave was incident on the surface of the composite film, reflection loss was first caused on the surface of the shield due to the impedance mismatch, which was mainly ascribed to the charge carriers that directly interact with the electromagnetic field on the surface of each layer of MXene nanosheets. Then, the transmitted electromagnetic waves continued to cause reflection loss through the next MXene layer. In addition, the electromagnetic wave entering the shielding material interacted with the charge of MXene or the magnetic dipole. Moreover, the internal reflection led to a large amount of polarization and loss at the interface or defect location to cause the absorption of the electromagnetic wave. In particular, the multilayer-structure ANF/MXene films were able to reflect and absorb multiple times inside the shield to attenuate or eliminate internal electromagnetic waves, resulting in an excellent EMI shielding performance. In order to more intuitively evaluate the shielding performance of the multilayer-structure film in this work, the SSE/t and thickness of the alternating film are compared with other electromagnetic shielding materials (Figure 4f and Table S2), such as metals and carbon-based materials. The multilayer-structure film not only shows a high SSE/t value of 4718 dB·cm^2^ g^−1^ and ultra-thin thickness of only 0.03 mm; in addition, due to the existence of ANF, the composite film has high mechanical properties, which provide the possibility for more practical applications.

### 3.4. Thermal Insulation and Photothermal Conversion Performance of Alternating Laminated ANF/MXene Films

**Figure 5:**
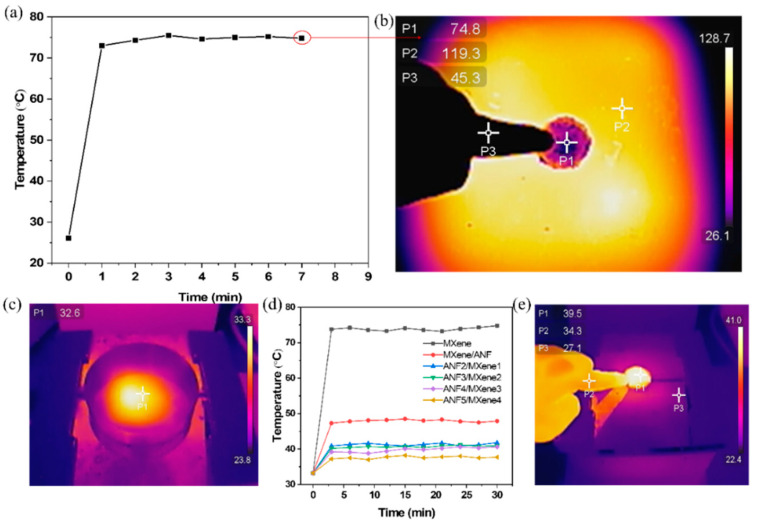
(**a**) Surface temperature curve of ANF5/MXene4 film at a temperature of 130 °C; (**b**) thermal image of ANF5/MXene4 film surface at a temperature of 130 °C; (**c**) thermal imaging under simulated sunlight; (**d**) surface temperature curves of multilayer-structure films under simulated sunlight; (**e**) thermal imaging of ANF5/MXene4 film surface under simulated sunlight.

Multilayer-structure film not only presents excellent mechanical properties and EMI shielding performance but also shows potential applications in thermal insulation. The thermogravimetric curves of different films at a temperature of 30–800 °C are shown in Figure S7. The decomposition temperature of ANF was about 500 °C, which was consistent with the previous report [56], indicating excellent thermal stability. Therefore, the multilayer-structure film provides possibilities for the design of many advanced functional materials, such as insulation materials and high-temperature-resistant devices. Figure 5a shows the surface-temperature change of the ANF5/MXene4 film (100 μm) placed on a stable heat source at a temperature of 130 °C. Due to the low thermal conductivity and excellent thermal stability of ANF [43], within several seconds, the surface temperature of the film rose to 73 °C; then, the temperature was maintained a few minutes instead of continuously rising. The thermal image of the film after heating for seven minutes under the heat source is shown in Figure 5b. The surface temperature of the film was maintained at about 74.8 °C, while the subface of the film was around 119.3 °C, indicating great heat insulation. In addition, the temperature change in the film under sunlight was tested by simulated sunlight. Figure 5c shows the temperature of the area under sunlight (about 32.6 °C). Due to MXene exhibiting an absorption efficiency of more than 90% under the solar spectrum [57,58], the surface temperature of the film reached above 70 °C under the simulated sunlight (Figure 5d and Figure S8), which provides for potential applications in photothermal conversion. However, in some practical applications, the high temperature might be adverse to functional devices. Interestingly, when the multilayer film was placed under the simulated light source, the surface temperature of the film only increased slightly at the beginning and remained basically stable within half an hour. ANF as a protective layer isolates a large part of the incident light [59]. For the ANF/MXene series of films, the ANF on the top layer blocks most of the sunlight. In addition, the surface temperature of the multilayer film decreases slightly with the increase in the number of layers. This occurs because with the increase in the number of layers of film, it becomes more and more difficult for the MXene at the bottom to absorb sunlight. While a small part of the incident light enters into contact with MXene to produce light-to-heat conversion, the heat is transferred to the entire film to slightly increase the temperature of the film surface (Figure 5d and Figure S8). The surface of the film could then be gently touched by fingers, leaving only a slight, soft impression (Figure 5e). Therefore, the multilayer composite films can be used in many situations, such as used in flexible circuit boards to provide interference shielding and blocking of heat generation or to protect small mobile devices from radiation under outdoor light.

## 4. Conclusions

In summary, ultra-thin, lightweight multilayer-structure ANF/MXene EMI shielding film with strong mechanical properties was successfully prepared by a simple AVAF process. Via the effective combination of the strong mechanical properties of ANF and the ultra-high conductive network of MXene forming a multilayer structure, the multilayer-structure film ANF2/MXene1 with a thickness of 30 μm presents a high tensile strength of 177.7 MPa. In addition, ANF5/MXene4 exhibits an EMI shielding efficiency of 37.5 dB and a high EMI SSE/t of 4718 dB·cm^2^ g^−1^, which is much higher than the 23.8 dB of homogeneous MXene/ANF film. Moreover, the multilayer-structure film also shows excellent thermal insulation properties and features enabling avoidance of light-to-heat conversion. This simple and versatile composite EMI shielding film has potential significance in practical applications, such as next-generation smart electronic equipment, communications, and military applications.

## Data Availability

Not applicable.

## Supplementary Materials

The following are available online at https://www.mdpi.com/article/10.3390/nano11113041/s1, Figure S1: (a,b) SEM images of Ti_3_AlC_2_ and m-Ti_3_C_2_T_x_, respectively; Figure S2: XRD patterns of Ti_3_AlC_2_ and d-Ti_3_C_2_T_x_; Figure S3: (a) XPS spectrum of MXene nanosheets. (b) O 1s spectra of the MXene nanosheets; Figure S4: Diagram of the transformation process of ANF nanofibers (7 days); Figure S5: Tyndall effect diagram of Ti_3_C_2_T_x_ and ANF solutions, respectively; Figure S6: (a,b) SEM image of the surface and cross-section of the homogeneously mixed MXene/ANF film; Figure S7: Thermogravimetric curve of MXene, ANF and alternating laminated ANF/MXene films; Figure S8: (a–f) Thermal image of MXene, homogeneously mixed MXene/ANF and alternating laminated films under the simulated sunlight; Table S1: The mechanical properties of alternately laminated ANF/MXene film and other EMI shielding materials are compared; Table S2: Performance comparison between alternating laminated ANF/MXene film and other EMI shielding materials.

## References

[1] Shahzad F., Alhabeb M., Hatter C.B., Anasori B., Hong S.M., Koo C.M., Gogotsi Y. (2016). Electromagnetic interference shielding with 2D transition metal carbides (MXenes). Science.

[2] Yun T., Kim H.T., Iqbal A., Cho Y.S., Lee G.S., Kim M., Kim S.J., Kim D., Gogotsi Y., Kim S.O. (2020). Electromagnetic Interference Shielding: Electromagnetic Shielding of Monolayer MXene Assemblies. Adv. Mater..

[3] Xiang C., Guo R., Lin S., Jiang S., Lan J., Wang C., Cui C., Xiao H., Zhang Y. (2019). Lightweight and ultrathin TiO_2_-Ti_3_C_2_T_X_/graphene film with electromagnetic interference shielding. Chem. Eng. J..

[4] Li H., Yuan D., Li P., He C. (2019). High conductive and mechanical robust carbon nanotubes/waterborne polyurethane composite films for efficient electromagnetic interference shielding. Compos. Part A Appl. Sci. Manuf..

[5] Song Q., Ye F., Yin X., Li W., Li H., Liu Y., Li K., Xie K., Li X., Fu Q. (2017). Carbon Nanotube-Multilayered Graphene Edge Plane Core-Shell Hybrid Foams for Ultrahigh-Performance Electromagnetic-Interference Shielding. Adv. Mater..

[6] Weng G.-M., Li J., Alhabeb M., Karpovich C., Wang H., Lipton J., Maleski K., Kong J., Shaulsky E., Elimelech M. (2018). Layer-by-Layer Assembly of Cross-Functional Semi-transparent MXene-Carbon Nanotubes Composite Films for Next-Generation Electromagnetic Interference Shielding. Adv. Funct. Mater..

[7] Anand S., Pauline S. (2020). Electromagnetic Interference Shielding Properties of BaCo_2_Fe_16_O_27_ Nanoplatelets and RGO Reinforced PVDF Polymer Composite Flexible Films. Adv. Mater. Interfaces.

[8] Lan C., Li C., Hu J., Yang S., Qiu Y., Ma Y. (2018). High-Loading Carbon Nanotube/Polymer Nanocomposite Fabric Coatings Obtained by Capillarity-Assisted “Excess Assembly” for Electromagnetic Interference Shielding. Adv. Mater. Interfaces.

[9] Liang C., Qiu H., Han Y., Gu H., Song P., Wang L., Kong J., Cao D., Gu J. (2019). Superior electromagnetic interference shielding 3D graphene nanoplatelets/reduced graphene oxide foam/epoxy nanocomposites with high thermal conductivity. J. Mater. Chem. C.

[10] Ma Z., Kang S., Ma J., Shao L., Zhang Y., Liu C., Wei A., Xiang X., Wei L., Gu J. (2020). Ultraflexible and Mechanically Strong Double-Layered Aramid Nanofiber–Ti_3_C_2_T_x_ MXene/Silver Nanowire Nanocomposite Papers for High-Performance Electromagnetic Interference Shielding. ACS Nano.

[11] Jia L.-C., Yan D.-X., Liu X., Ma R., Wu H.-Y., Li Z.-M. (2018). Highly Efficient and Reliable Transparent Electromagnetic Interference Shielding Film. ACS Appl. Mater. Interfaces.

[12] Choi H.Y., Lee T.-W., Lee S.-E., Lim J., Jeong Y.G. (2017). Silver nanowire/carbon nanotube/cellulose hybrid papers for electrically conductive and electromagnetic interference shielding elements. Compos. Sci. Technol..

[13] Shen B., Li Y., Zhai W., Zheng W. (2016). Compressible Graphene-Coated Polymer Foams with Ultralow Density for Adjustable Electromagnetic Interference (EMI) Shielding. ACS Appl. Mater. Interfaces.

[14] Lu D., Mo Z., Liang B., Yang L., He Z., Zhu H., Tang Z., Gui X. (2018). Flexible, lightweight carbon nanotube sponges and composites for high-performance electromagnetic interference shielding. Carbon.

[15] Hantanasirisakul K., Gogotsi Y. (2018). Electronic and Optical Properties of 2D Transition Metal Carbides and Nitrides (MXenes). Adv. Mater..

[16] Naguib M., Kurtoglu M., Presser V., Lu J., Niu J., Heon M., Hultman L., Gogotsi Y., Barsoum M.W. (2011). Two-Dimensional Nanocrystals Produced by Exfoliation of Ti_3_AlC_2_. Adv. Mater..

[17] Cao M.-S., Cai Y.-Z., He P., Shu J.-C., Cao W.-Q., Yuan J. (2019). 2D MXenes: Electromagnetic property for microwave absorption and electromagnetic interference shielding. Chem. Eng. J..

[18] Fan Z., Wang Y., Xie Z., Wang D., Yuan Y., Kang H., Su B., Cheng Z., Liu Y. (2018). Modified MXene/Holey Graphene Films for Advanced Supercapacitor Electrodes with Superior Energy Storage. Adv. Sci..

[19] Li X., Hao J., Liu R., He H., Wang Y., Liang G., Liu Y., Yuan G., Guo Z. (2020). Interfacing MXene flakes on fiber fabric as an ultrafast electron transport layer for high performance textile electrodes. Energy Storage Mater..

[20] Nan J., Guo X., Xiao J., Li X., Chen W., Wu W., Liu H., Wang Y., Wu M., Wang G. (2019). Nanoengineering of 2D MXene-Based Materials for Energy Storage Applications. Small.

[21] Zhang X., Zhang Z., Zhou Z. (2018). MXene-based materials for electrochemical energy storage. J. Energy Chem..

[22] Wang Q.-W., Zhang H.-B., Liu J., Zhao S., Xie X., Liu L., Yang R., Koratkar N., Yu Z.-Z. (2018). Multifunctional and Water-Resistant MXene-Decorated Polyester Textiles with Outstanding Electromagnetic Interference Shielding and Joule Heating Performances. Adv. Funct. Mater..

[23] Jiang Q., Wu C., Wang Z., Wang A.C., He J.-H., Wang Z.L., Alshareef H.N. (2018). MXene electrochemical microsupercapacitor integrated with triboelectric nanogenerator as a wearable self-charging power unit. Nano Energy.

[24] Liang C., Qiu H., Song P., Shi X., Kong J., Gu J. (2020). Ultra-light MXene aerogel/wood-derived porous carbon composites with wall-like “mortar/brick” structures for electromagnetic interference shielding. Sci. Bull..

[25] Natu V., Hart J.L., Sokol M., Chiang H., Taheri M.L., Barsoum M.W. (2019). Edge Capping of 2D-MXene Sheets with Polyanionic Salts To Mitigate Oxidation in Aqueous Colloidal Suspensions. Angew. Chem..

[26] Jia X., Shen B., Zhang L., Zheng W. (2021). Corrigendum to “Construction of compressible Polymer/MXene composite foams for high-performance absorption-dominated electromagnetic shielding with ultra-low reflectivity”. Carbon.

[27] Zhou B., Li Q., Xu P., Feng Y., Ma J., Liu C., Shen C. (2021). Asymmetric Sandwich Structural Cellulose-based Film with Self-supported MXene and AgNW Layers for Flexible Electromagnetic Interference Shielding and Thermal Management. Nanoscale.

[28] Xu H., Yin X., Li X., Li M., Liang S., Zhang L., Cheng L. (2019). Lightweight Ti_2_CT_x_ MXene/Poly(vinyl alcohol) Composite Foams for Electromagnetic Wave Shielding with Absorption-Dominated Feature. ACS Appl. Mater. Interfaces.

[29] Zhao S., Zhang H.-B., Luo J.-Q., Wang Q.-W., Xu B., Hong S., Yu Z.-Z. (2018). Highly Electrically Conductive Three-Dimensional Ti_3_C_2_T_x_ MXene/Reduced Graphene Oxide Hybrid Aerogels with Excellent Electromagnetic Interference Shielding Performances. ACS Nano.

[30] Monastyreckis G., Mishnaevsky L., Hatter C.B., Aniskevich A., Gogotsi Y., Zeleniakiene D. (2020). Micromechanical modeling of MXene-polymer composites. Carbon.

[31] Lee G.S., Yun T., Kim H., Kim I.H., Choi J., Lee S.H., Lee H.J., Hwang H.S., Kim J.G., Kim D. (2020). Mussel Inspired Highly Aligned Ti_3_C_2_T_x_ MXene Film with Synergistic Enhancement of Mechanical Strength and Ambient Stability. ACS Nano.

[32] Sun R., Zhang H.-B., Liu J., Xie X., Yang R., Li Y., Hong S., Yu Z.-Z. (2017). Highly Conductive Transition Metal Carbide/Carbonitride(MXene)@polystyrene Nanocomposites Fabricated by Electrostatic Assembly for Highly Efficient Electromagnetic Interference Shielding. Adv. Funct. Mater..

[33] Huang J., Wang T., Su Y., Ding Y., Tu C., Li W. (2021). Hydrophobic MXene/Hydroxyethyl Cellulose/Silicone Resin Composites with Electromagnetic Interference Shielding. Adv. Mater. Interfaces.

[34] Cao W.-T., Chen F.-F., Zhu Y.-J., Zhang Y.-G., Jiang Y.-Y., Ma M.-G., Chen F. (2018). Binary Strengthening and Toughening of MXene/Cellulose Nanofiber Composite Paper with Nacre-Inspired Structure and Superior Electromagnetic Interference Shielding Properties. ACS Nano.

[35] Sun Y., Ding R., Hong S.Y., Lee J., Seo Y.-K., Nam J.-D., Suhr J. (2021). MXene-xanthan nanocomposite films with layered microstructure for electromagnetic interference shielding and Joule heating. Chem. Eng. J..

[36] Zhou B., Zhang Z., Li Y., Han G., Feng Y., Wang B., Zhang D., Ma J., Liu C. (2020). Flexible, Robust, and Multifunctional Electromagnetic Interference Shielding Film with Alternating Cellulose Nanofiber and MXene Layers. ACS Appl. Mater. Interfaces.

[37] Naeimirad M., Zadhoush A., Neisiany R.E., Ramakrishna S., Salimian S., Leal A.A. (2018). Influence of microfluidic flow rates on the propagation of nano/microcracks in liquid core and hollow fibers. Theor. Appl. Fract. Mech..

[38] Esmaeely Neisiany R., Nouri Khorasani S., Kong Yoong Lee J., Razavi J., Enayati M.S., Naeimirad M., Berto F., Ramakrishna S. (2019). Core-shell nanofibers for developing self-healing materials: Recent progress and future directions. Mater. Des. Process. Commun..

[39] Yang M., Cao K., Sui L., Qi Y., Zhu J., Waas A., Arruda E.M., Kieffer J., Thouless M.D., Kotov N.A. (2011). Dispersions of Aramid Nanofibers: A New Nanoscale Building Block. ACS Nano.

[40] Yang B., Wang L., Zhang M., Luo J., Ding X. (2019). Timesaving, High-Efficiency Approaches To Fabricate Aramid Nanofibers. ACS Nano.

[41] Wu Y., Wang F., Huang Y. (2018). Facile and simple fabrication of strong, transparent and flexible aramid nanofibers/bacterial cellulose nanocomposite membranes. Compos. Sci. Technol..

[42] Xie C., He L., Shi Y., Guo Z.-X., Qiu T., Tuo X. (2019). From Monomers to a Lasagna-like Aerogel Monolith: An Assembling Strategy for Aramid Nanofibers. ACS Nano.

[43] Ma T., Zhao Y., Ruan K., Liu X., Zhang J., Guo Y., Yang X., Kong J., Gu J. (2019). Highly Thermal Conductivities, Excellent Mechanical Robustness and Flexibility, and Outstanding Thermal Stabilities of Aramid Nanofiber Composite Papers with Nacre-Mimetic Layered Structures. ACS Appl. Mater. Interfaces.

[44] Yang B., Zhang M., Lu Z., Tan J., Luo J., Song S., Ding X., Wang L., Lu P., Zhang Q. (2019). Comparative study of aramid nanofiber (ANF) and cellulose nanofiber (CNF). Carbohydr. Polym..

[45] Alhabeb M., Maleski K., Anasori B., Lelyukh P., Clark L., Sin S., Gogotsi Y. (2017). Guidelines for Synthesis and Processing of Two-Dimensional Titanium Carbide (Ti_3_C_2_T_x_ MXene). Chem. Mater..

[46] Yang B., Wang L., Zhang M., Luo J., Lu Z., Ding X. (2020). Fabrication, Applications, and Prospects of Aramid Nanofiber. Adv. Funct. Mater..

[47] Raagulan K., Kim B.M., Chai K.Y. (2020). Recent Advancement of Electromagnetic Interference (EMI) Shielding of Two Dimensional (2D) MXene and Graphene Aerogel Composites. Nanomaterials.

[48] Lei C., Zhang Y., Liu D., Wu K., Fu Q. (2020). Metal-Level Robust, Folding Endurance, and Highly Temperature-Stable MXene-Based Film with Engineered Aramid Nanofiber for Extreme-Condition Electromagnetic Interference Shielding Applications. ACS Appl. Mater. Interfaces.

[49] Tian W., VahidMohammadi A., Reid M.S., Wang Z., Ouyang L., Erlandsson J., Pettersson T., Wågberg L., Beidaghi M., Hamedi M.M. (2019). MXene Nanocomposites: Multifunctional Nanocomposites with High Strength and Capacitance Using 2D MXene and 1D Nanocellulose (Adv. Mater. 41/2019). Adv. Mater..

[50] Zhang H., Wang L., Chen Q., Li P., Zhou A., Cao X., Hu Q. (2016). Corrigendum to “Preparation, mechanical and anti-friction performance of MXene/polymer composites”. Mater. Des..

[51] Cao W., Ma C., Tan S., Ma M., Wan P., Chen F. (2019). Ultrathin and Flexible CNTs/MXene/Cellulose Nanofibrils Composite Paper for Electromagnetic Interference Shielding. Nano-Micro Lett..

[52] Chung D.D.L. (2001). Electromagnetic interference shielding effectiveness of carbon materials. Carbon.

[53] Wen B., Cao M., Lu M., Cao W., Shi H., Liu J., Wang X., Jin H., Fang X., Wang W. (2014). Reduced Graphene Oxides: Light-Weight and High-Efficiency Electromagnetic Interference Shielding at Elevated Temperatures. Adv. Mater..

[54] Liang L., Li Q., Yan X., Feng Y., Wang Y., Zhang H.-B., Zhou X., Liu C., Shen C., Xie X. (2021). Multifunctional Magnetic Ti_3_C_2_T_x_ MXene/Graphene Aerogel with Superior Electromagnetic Wave Absorption Performance. ACS Nano.

[55] Naguib M., Mashtalir O., Carle J., Presser V., Lu J., Hultman L., Gogotsi Y., Barsoum M.W. (2012). Two-Dimensional Transition Metal Carbides. ACS Nano.

[56] Li R., Zhang L., Shi L., Wang P. (2017). MXene Ti3C2: An Effective 2D Light-to-Heat Conversion Material. ACS Nano.

[57] Wang L., Zhang M., Yang B., Tan J., Ding X. (2020). Highly Compressible, Thermally Stable, Light-Weight, and Robust Aramid Nanofibers/Ti3AlC2 MXene Composite Aerogel for Sensitive Pressure Sensor. ACS Nano.

[58] Zhou B., Su M., Yang D., Han G., Feng Y., Wang B., Ma J., Ma J., Liu C., Shen C. (2020). Flexible MXene/Silver Nanowire-Based Transparent Conductive Film with Electromagnetic Interference Shielding and Electro-Photo-Thermal Performance. ACS Appl. Mater. Interfaces.

[59] Cheng Z., Hong D., Dai Y., Jiang C., Meng C., Luo L., Liu X. (2018). Highly improved UV resistance and composite interfacial properties of aramid fiber via iron (III) coordination. Appl. Surf. Sci..

