# Supplementary material for: Mechanically Robust Flexible Multilayer Aramid Nanofibers and MXene Film for High-Performance Electromagnetic Interference Shielding and Thermal Insulation
Source: Nanomaterials (Basel). 2021 Nov 12;11(11):3041. doi: 10.3390/nano11113041 (PMC8620062; doi:10.3390/nano11113041)
Supplement: Supplementary file 1 [file nanomaterials-11-03041-s001.zip › nanomaterials-1449795-supplementary.pdf]

nanomaterials

Supplementary Materials

Mechanically Robust Flexible Multilayer Aramid Nanofibers and MXene Film for High-Performance Electromagnetic Interference Shielding and Thermal Insulation

Jun Zhou [1,2], Junsheng Yu [2], Dongyu Bai [1,3,*], Huili Liu [3,4] and Lu Li [1,2,*]

1 Chongqing Key Laboratory of Materials Surface & Interface Science, School of Materials Science and Engineering, Chongqing University of Arts and Sciences, Chongqing 402160, China; along_zj@163.com
2 State Key Laboratory of Electronic Thin Films and Integrated Devices, School of Optoelectronic Science and Engineering, University of Electronic Science and Technology of China (UESTC), Chengdu 610054, China; jsyu@uestc.edu.cn
3 School of Chemistry & Chemical Engineering, Chongqing University, Chongqing 400044, China; liuhl0827@163.com
4 Chongqing Key Laboratory of Environmental Materials and Remediation Technologies, College of Chemistry and Environmental Engineering, Chongqing University of Arts and Sciences, Chongqing 402160, China
* Correspondence: dongyu_bai@163.com or dongyu_bai@cqwu.edu.cn (D.B.); lli@cqwu.edu.cn (L.L.)

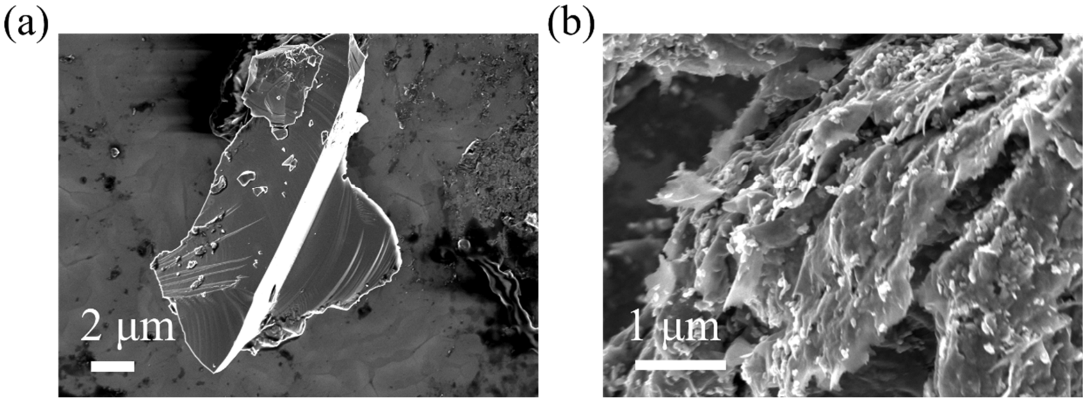

Figure S1. (**a**,**b**) SEM images of Ti_3AlC_2 and m-$Ti_3C_2T_x$, respectively.

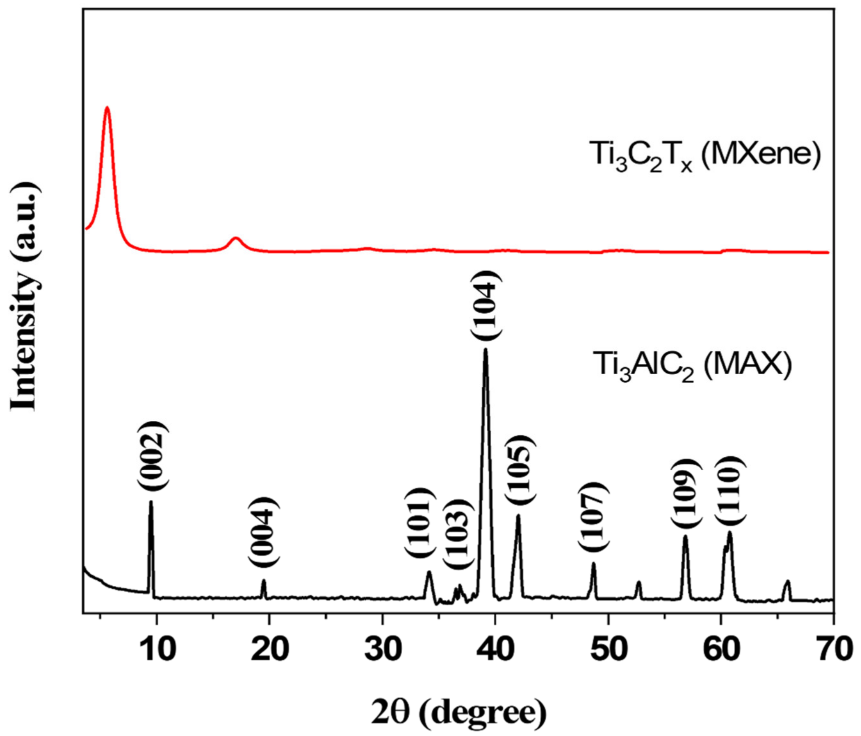

Figure S2. XRD patterns of Ti_3AlC_2 and d-$Ti_3C_2T_x$.

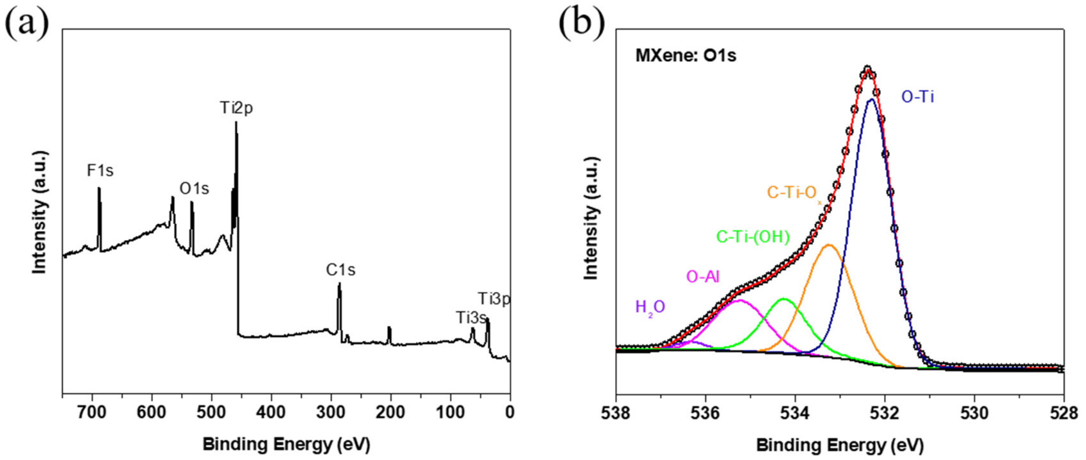

Figure S3. (**a**) XPS spectrum of MXene nanosheets. (**b**) O 1s spectra of the MXene nanosheets.

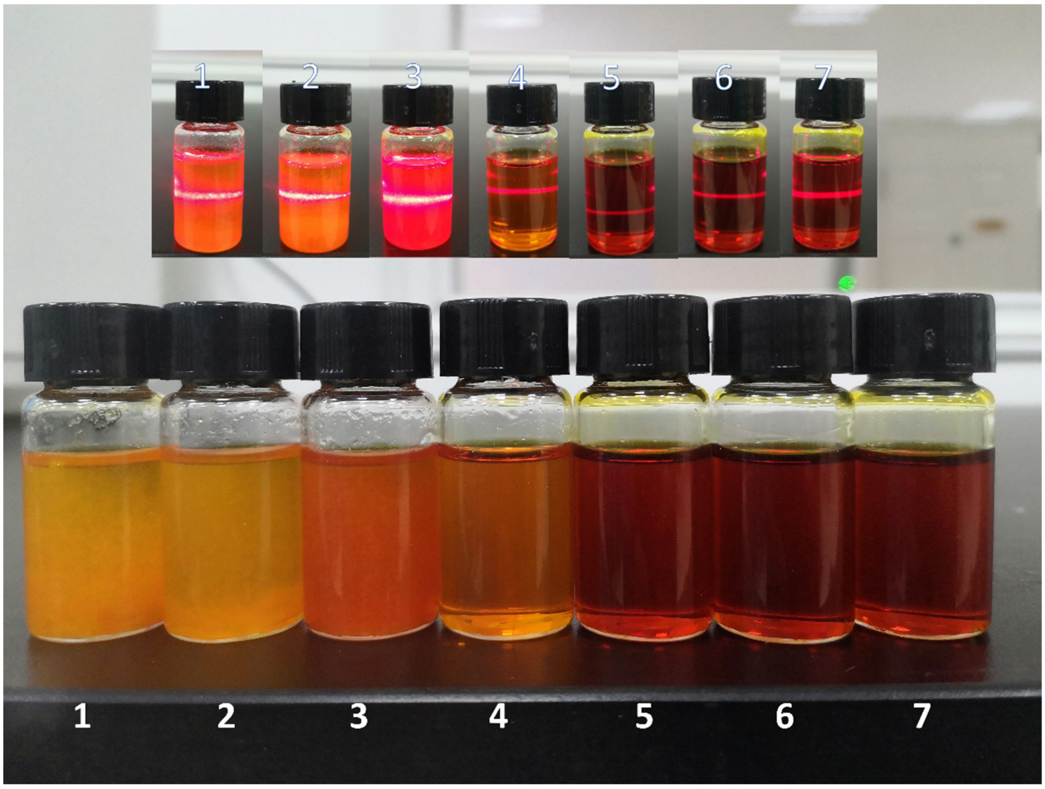

Figure S4. Diagram of the transformation process of ANF nanofibers (7 days).

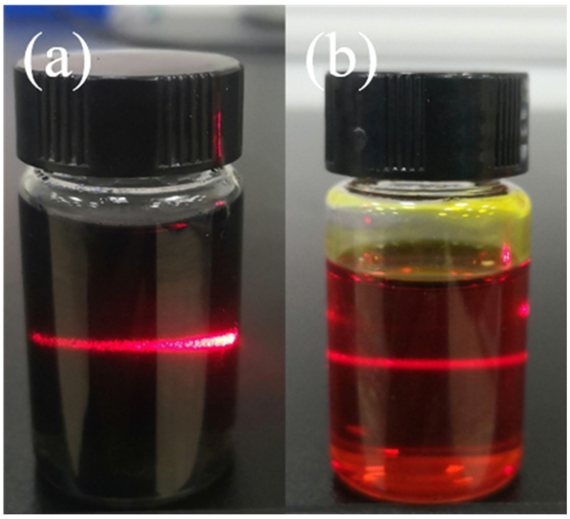

Figure S5. (**a**,**b**) Tyndall effect diagram of $Ti_3C_2T_x$ and ANF solutions, respectively.

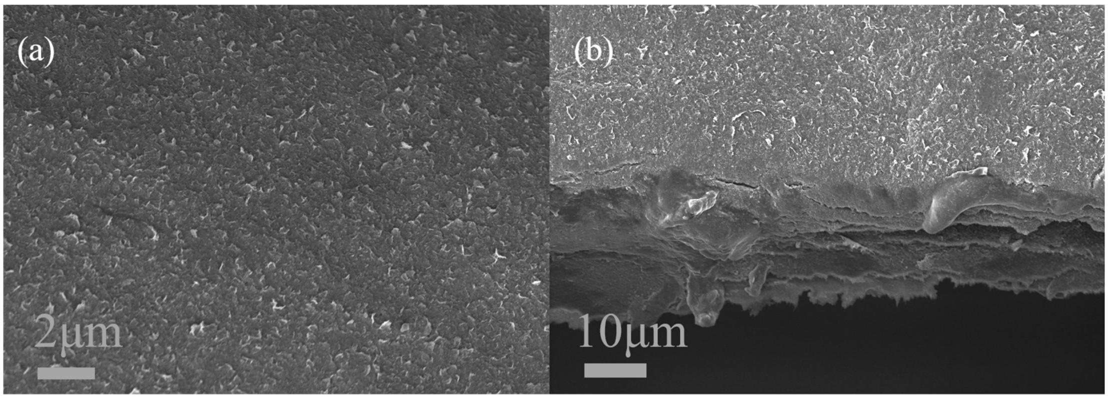

Figure S6. (**a**,**b**) SEM image of the surface and cross-section of the homogeneously mixed MXene/ANF film.

Table S1. The mechanical properties of alternately laminated ANF/MXene film and other EMI shielding materials are compared.

Materials	EMI	Tensile Strain	Fracture Strain	Refs
CNTs/MXene/CNF	38	97.9	4.6	[51]
CNF/MXene	40	112.5	4.3	[36]
PEDOT:PSS/MXene	42	30.18	1.51	S1
AgNW/MXene	35	63.8	1.29	S2
CNT/$CoFe_2O_4$	30	30	4	S3
MWCNTs/ Fe3O4/PLA	22	17.5	2.8	S4
This work	37.5	177.7	10.13	

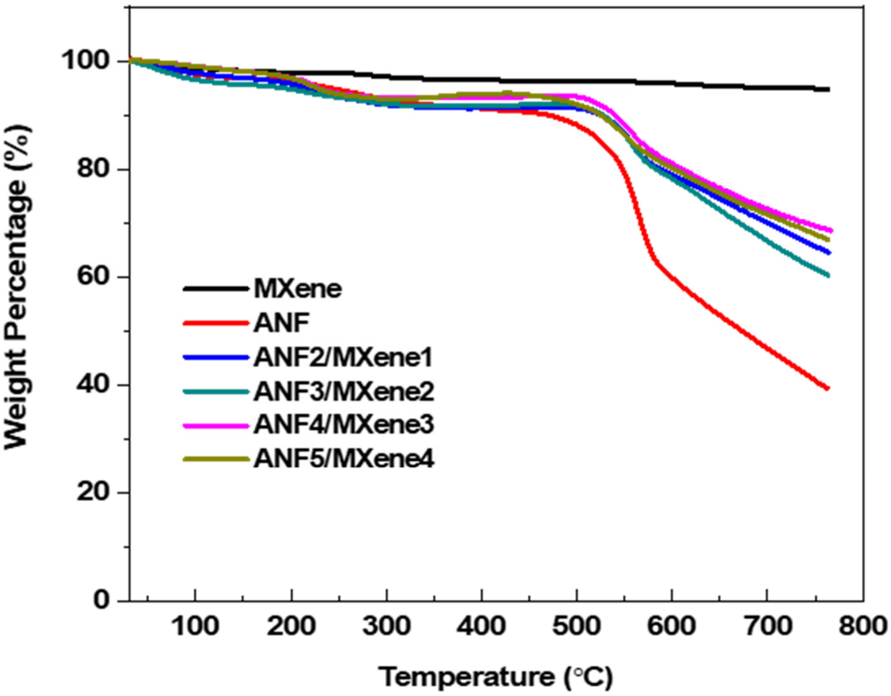

Figure S7. Thermogravimetric curve of MXene, ANF and alternating laminated ANF/MXene films.

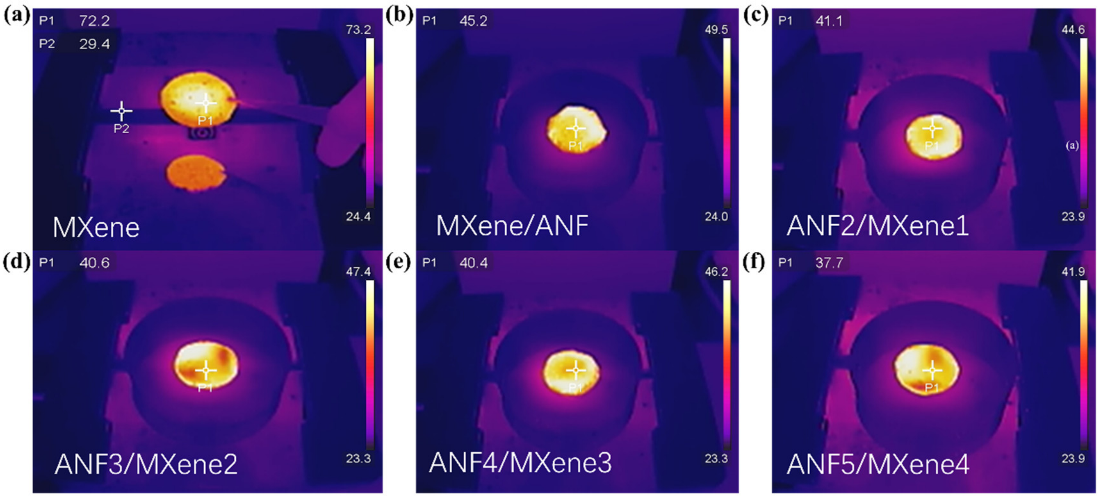

Figure S8. (**a**–**f**) Thermal image of MXene, homogeneously mixed MXene/ANF and alternating laminated films under the simulated sunlight.

Table S2. Performance comparison between alternating laminated ANF/MXene film and other EMI shielding materials.

**Type**	**Materials**	**Thickness (mm)**	**EMI SE (dB)**	**SSE/t (dB cm^2 g^{-1})**	**Refs**
	AgNW	0.5	35	2416	S5
	CF/Ni	0.31	72.7	1376	S6
Metal-bsaed	CuNi	1.5	54.6	1580	S7
	Cmf-Au-GIO/PDMS	2	30.5	1314.7	S8
	rGO/Fe3O4	2.5	18	176	S9
	rGO	2.5	45.1	692	S10
	rGO/PEI	2.3	10	152.2	S11
	rGO/PMMA	0.24	19	1042	S12
Carbon-based	Graphene/PDMS	0.1	20	3330	S13
	CNF mat	2.9	52.2	1361.6	S14
	CNT-sponge	2.4	22	4583	S15
	MWCNT/CNF	0.15	46.4	4017.3	S16
	MWCNT/PS	2	30	285	S17
	MWCNT-PEO	0.15	35	1372.5	S18
	MXene/rGO	2	56.4	9400	[28]
MXene-based	MXene/PS	2	62	255.2	[31]
	MXene/PEDOT:PSS	0.015	9	3636	S1
	MXene/CNF	0.167	25	1326	[33]
	MXene/CNT	0.04	23.4	5219	[51]
This work	ANF5/MXene4	0.03	37.5	4718	
	ANF4/MXene3	0.03	34.8	4370	

References

1. R. Liu, M. Miao, Y. Li, J. Zhang, S. Cao, X. Feng. Ultrathin Biomimetic Polymeric Ti3C2Tx MXene Composite Films for Electromagnetic Interference Shielding. *ACS Appl. Mater. Interfaces* **2018**, *10*, 44787–44795.
2. M. Miao, R. Liu, S. Thaiboonrod, L. Shi, S. Cao, J. Zhang, J. Fang, X. Feng. Silver nanowires intercalating Ti3C2Tx MXene composite films with excellent flexibility for electromagnetic interference shielding. *J. Mater. Chem. C* **2020**, *8*, 3120–3126.
3. G.-H. Lim, S. Woo, H. Lee, K.-S. Moon, H. Sohn, S.-E. Lee, B. Lim. Mechanically Robust Magnetic Carbon Nanotube Papers Prepared with CoFe2O4 Nanoparticles for Electromagnetic Interference Shielding and Magnetomechanical Actuation. *ACS Appl. Mater. Interfaces* **2017**, *9*, 40628–40637.
4. S. Liu, P. Wang, Y. Yang, Y. Yang, G. Wu, H. Li, K. Wang, B. Wang, M. Liu, Y. Zhang, J. Wu, J. Zhang, Y. Jing, F. Li, M. Zhang. Influence of MWCNTs and nano-Fe(3)O(4) on the properties and structure of MWCNTs/Fe3O4/PLA composite film with electromagnetic interference shielding function. *J. Polym. Res.* **2020**, *288*.
5. J. Ma, K. Wang, M. Zhan. A comparative study of structure and electromagnetic interference shielding performance for silver nanostructure hybrid polyimide foams. *RSC Adv.* **2015**, *5*, 65283–65296.
6. D. Xing, L. Lu, K. S. Teh, Z. Wan, Y. Xie, Y. Tang. Highly flexible and ultra-thin Ni-plated carbon-fabric/polycarbonate film for enhanced electromagnetic interference shielding. *Carbon* **2018**, *132*, 32–41.
7. K. Ji, H. Zhao, J. Zhang, J. Chen, Z. Dai. Fabrication and electromagnetic interference shielding performance of open-cell foam of a Cu–Ni alloy integrated with CNTs. *Appl. Surf. Sci.* **2014**, *311*, 351–356.
8. Y. Sun, S. Luo, H. Sun, W. Zeng, C. Ling, D. Chen, V. Chan, K. Liao. Engineering closed-cell structure in lightweight and flexible carbon foam composite for high-efficient electromagnetic interference shielding. *Carbon* **2018**, *136*, 299–308.
9. B. Shen, W. Zhai, M. Tao, J. Ling, W. Zheng. Lightweight, Multifunctional Polyetherimide/Graphene@Fe3O4 Composite Foams for Shielding of Electromagnetic Pollution. *ACS Appl. Mater. Interfaces* **2013**, *5*, 11383–11391.
10. D.-X. Yan, H. Pang, B. Li, R. Vajtai, L. Xu, P.-G. Ren, J.-H. Wang, Z.-M. Li. Structured Reduced Graphene Oxide/Polymer Composites for Ultra-Efficient Electromagnetic Interference Shielding. *Adv. Funct. Mater.* **2014**, *25*, 559–566.
11. J. Ling, W. Zhai, W. Feng, B. Shen, J. Zhang, W. ge Zheng. Facile Preparation of Lightweight Microcellular Polyetherimide/Graphene Composite Foams for Electromagnetic Interference Shielding. *ACS Appl. Mater. Interfaces* **2013**, *5*, 2677–2684.
12. H.-B. Zhang, Q. Yan, W.-G. Zheng, Z. He, Z.-Z. Yu. Tough Graphene–Polymer Microcellular Foams for Electromagnetic Interference Shielding. *ACS Appl. Mater. Interfaces* **2011**, *3*, 918–924.
13. Z. Chen, C. Xu, C. Ma, W. Ren, H.-M. Cheng. Lightweight and Flexible Graphene Foam Composites for High-Performance Electromagnetic Interference Shielding. *Adv. Mater.* **2013**, *25*, 1296–1300.
14. X. Hong, D. D. L. Chung. Carbon nanofiber mats for electromagnetic interference shielding. *Carbon* **2017**, *111*, 529–537.
15. M. Crespo, M. González, A. L. Elías, L. Pulickal Rajukumar, J. Baselga, M. Terrones, J. Pozuelo. Ultra-light carbon nanotube sponge as an efficient electromagnetic shielding material in the GHz range. *Phys Status Solidi-R* **2014**, *8*, 698–704.
16. H. Zhang, X. Sun, Z. Heng, Y. Chen, H. Zou, M. Liang. Robust and Flexible Cellulose Nanofiber/Multiwalled Carbon Nanotube Film for High-Performance Electromagnetic Interference Shielding. *Ind. Eng. Chem. Res.* **2018**, *57*, 17152–17160.
17. M. Arjmand, T. Apperley, M. Okoniewski, U. Sundararaj. Comparative study of electromagnetic interference shielding properties of injection molded versus compression molded multi-walled carbon nanotube/polystyrene composites. *Carbon* **2012**, *50*, 5126-5134.
18. L.-Q. Zhang, B. Yang, J. Teng, J. Lei, D.-X. Yan, G.-J. Zhong, Z.-M. Li. Tunable electromagnetic interference shielding effectiveness via multilayer assembly of regenerated cellulose as a supporting substrate and carbon nanotubes/polymer as a functional layer. *J. Mater. Chem. C* **2017**, *5*, 3130–3138.
